# Stress and Psychological Distress in Emerging Adulthood: A Gender Analysis

**DOI:** 10.3390/jcm9092859

**Published:** 2020-09-04

**Authors:** M. Pilar Matud, Amelia Díaz, Juan Manuel Bethencourt, Ignacio Ibáñez

**Affiliations:** 1 Department of Clinical Psychology, Psychobiology, and Methodology, Universidad de La Laguna, 38200 San Cristobal de La Laguna, Spain; jmbethen@ull.edu.es (J.M.B.); iibanez@ull.edu.es (I.I.); 2 Department of Personality, Assessment and Psychological Treatments, Universidad de Valencia, 46010 Valencia, Spain; Amelia.Diaz@uv.es

**Keywords:** emerging adulthood, psychological distress, stress, coping styles, social support, self-esteem

## Abstract

Emerging adulthood is a critical period of life that entails many life transitions in living arrangements, relationships, education and employment, which can generate stress and psychological distress in the emerging adult. The aim of the present study was to assess the relevance of stress, coping styles, self-esteem and perceived social support in the distress of emerging adult women and men. The sample consists of 4816 people (50% females) from the Spanish general population, ranging in age from 18 to 29 years old. All participants were assessed through questionnaires and scales that assess psychological distress, stress, coping styles, self-esteem and social support. Women scored higher than men in psychological distress, chronic stress, minor daily hassles, emotional coping style and social support, whereas men scored higher than women in rational and detachment coping styles and in self-esteem. Psychological distress was significantly predicted in women and men by high emotional coping style, lower self-esteem, high number of life events, and less social support. Another statistically significant predictor in men was less detachment coping style, whereas in women it was high chronic stress. The results of this research are relevant to healthcare professionals interested in improving the mental health of the emerging adult.

## 1. Introduction

In recent decades the existence of a new life stage at ages 18–29, known as emerging adulthood, has been proposed [1,2]. It is a distinct period from adolescence and young adulthood that have its own demographic, subjective and identity-related characteristics [3]. This is considered a critical period of life [4], and is the most unstable period of the life span [1]. Although young people’s experience of emerging adulthood may differ across national, cultural and socioeconomic contexts [2,5], the rise of emerging adulthood is an international phenomenon that occurs in developed countries and is increasing in developing countries [5].

This period often entails many life transitions in living arrangements, relationships, education and employment [3,6]. It is a period of heightened instability in which young people experience a series of loving relationships and frequent job changes before making lasting decisions [1]. The important changes of this period generate instability and uncertainty, and a significant mental health risk [1,7]. Epidemiological studies in the USA show that the 12-month prevalence of any psychiatric disorder is greater than 40% in people aged 18–29 years, and is higher in people of any age range, especially for mood disorders, anxiety disorders, and substance misuse [1]. It has been observed that, in the USA, the rates of major depressive episode in the last year among people aged 18–25 years increased from 8.1% to 13.2% between 2005–2017, and serious psychological distress in the last month increased among young adults ages 18 to 25 between 2008 and 2017, which is a period increase larger among women than among men [8]. Mood disorders and anxiety disorders were also the most prevalent psychiatric disorders in people aged 20–34 years in Japan [1]. Further, according to the WHO World Mental Health International College Student project, 31.4% of first-year students in 19 colleges across 8 countries (Australia, Belgium, Germany, Mexico, Northern Ireland, South Africa, Spain, United States) screened positive for at least 1 common DSM-IV anxiety, mood, or substance disorder in the last 12 months [9].

Although, not all emerging adults experience difficulties during the transitions typical of this stage of life, life transitions can generate distress for emerging adults [10], and the distress accompanying such transitions poses a considerable threat to the well-being of the emerging adults [7]. Epidemiological research and population surveys have concluded that women report higher psychological distress that men [11,12,13,14,15,16,17], although such differences vary according to socioeconomic variables, historical and contextual factors [15,16,17,18], and age [15]. Psychological distress is also an important mental health problem among university students, and is highly associated with suicidal behavior, which is more frequent in female than in male university students [19]. 

Emerging adulthood involves major transitions in social roles and high levels of stress, which may affect health in later life [20]. Stress is considered one of the most impactful psychological phenomena regarding its consequences for mental and physical health [21], and distress has been regarded as a maladaptive internal response to stressors [22]. Stress can include stress responses and stressors [23], and psychological stress is defined as the result of a relationship between the individual and their environment, which is evaluated by the subject as threatening or overflowing with their resources and that endangers their well-being [24]. During emerging adulthood young women and men seem to face multiple stressors and high demands connected to life transitions and changes in relationships, work, education and place of residence. Discrete events that are normative during emerging adulthood, such as moving out of the family home, entering college, or beginning paid work can be highly stressful, perpetuating or exaggerating other chronic strains [25]. While, episodic stressful life events are acute, temporary, psychologically threatening experiences, chronic stress refers to enduring pressures and strains [26]. Minor daily hassles, or daily stressors, are defined as routine daily life challenges, such as the everyday concerns of work, commuting between work and home, or more unexpected small events that disrupt daily life, such as unexpected work deadlines or malfunctioning computers [27]. It has been suggested that, although minor, interruptions like these may have a more immediate effect on well-being than major life events [27].

Stress is an inevitable part of life [22,24], but research has shown individual variability in the aftermath of exposure to stressful events and in how effectively they deal with stress. Various factors have been hypothesized to moderate or mediate the connection between social stress and health-related outcomes, although coping and social support have received the most attention in the research literature [28,29]. Coping is a multidimensional construct that refers to the actions (thoughts and behaviors) that people use to manage the demands of stressful events [30], and there is evidence that the ways in which people cope with stress affect their physical, psychological and social well-being [31].

Review studies have shown that perceived emotional and instrumental social support have protective effects on mental health [32], and research conducted in several countries has reported that low social support has been associated with increased psychological distress both, in women and men [4,33,34], but most studies have focused on either adolescents or older adults [4]. Self-esteem is another factor that may be important in mental health in emerging adulthood. Self-esteem refers to the judgments individuals make about their own self-worth [28], and it is considered a mechanism by which stressors impact mental health [28,29,35,36]. Moreover, research has reported that low self-esteem predicts depression in young adulthood [37], and is a risk factor for depressive symptoms throughout all phases of adult life [38].

Whereas emerging adulthood is regarded as a critical period of life [4] characterized by many life transitions and significant mental health risk [1,7,9,39], research on psychological distress in emerging adulthood is scarce and one of the important areas is examining how gender shapes experiences in emerging adulthood. Gender refers to socially constructed norms, behaviors, activities, relationships and attributes that society considers appropriate for women and men [40], and it is recognized as an important social determinant of health [40,41,42]. Given that emerging adulthood is characterized by identity issues in identifying oneself and what one wants to be, and it requires young men and women to explore their future possibilities, gender can play a crucial role in the ongoing development of one’s identity, as well as in relationships with romantic partners, friends and family, and in the implications of risk behaviors and in associated mental health outcomes [43].

Despite the relevance of gender in this period of life, there is no research, to our knowledge, that analyzes psychological distress and its association with stress, coping styles, self-esteem and social support from a gender perspective. Therefore, the aim of the present study was to assess the relevance of stress, coping styles, self-esteem and perceived social support in the distress of emerging adult women and men. A second aim was to examine the existence of differences between emerging adult men and women in distress, in stress (number of life events, chronic stress and minor daily hassles), in coping styles, in self-esteem and in perceived social support.

## 2. Materials and Methods

### 2.1. Participants

The study sample consisted of 4816 people, 50% women, from the Spanish general population, ranging in age from 18 to 29 years. The mean age for the male group was 23.02 years (*SD* = 3.31) and for the female group 22.93 (*SD* = 3.29 years), differences that were not statistically significant, *t* (4814) = 0.89, *p* = 0.37. All participants did not receive financial compensation for their participation. Access to the sample was through educational and work centers of different Spanish localities, as well as resorting to the social net of nursing, psychology and sociology university students from seven Spanish universities trained in administering the tests, who received course credits for this task. Criteria for inclusion in the study were: (1) to be between 18 and 29 years old; (2) to speak and understand Spanish correctly.

The study was conducted in accordance with the Declaration of Helsinki. All subjects gave their informed consent for inclusion before they participated in the study, and tests were individually and manually completed on a paper version. Ethical standards have been complied in the treatment of the sample and no names or any other data identifying the participant were used in the tests. This study is part of extensive research on gender and health and was positively evaluated by the Ethical Committee on Animal Research and Well-Being of the University of La Laguna (study approval number 2015-0170).

### 2.2. Data Collection

The collected data were considered as either outcome (dependent) or predictor (independent) variables to fit the study aim.

#### 2.2.1. Dependent Variable: Psychological Distress

Psychological distress was assessed by using the GHQ-28 [44]. The General Health Questionnaire (GHQ) is a self-administered screening questionnaire designed for detecting those with a diagnosable psychiatric disorder [45]. “It concerns itself with two major classes of phenomena: Inability to carry out one’s normal “healthy” functions, and the appearance of new phenomena of a distressing nature” [44] (p. 139). The GHQ-28 includes 28 items that evaluate somatic symptoms, anxiety and insomnia, social dysfunction and severe depression. Items were scored using the Likert-type scale from 0 “less than usual” to 3 “much more than usual”. In the present sample, the Cronbach alpha for the 28 items of the questionnaire was 0.95.

#### 2.2.2. Independent Variables: Stress, Coping Styles, Self-Esteem and Social Support

Stress was assessed with three questionnaires: (1) Life Events Questionnaire [46] that includes 22 items evaluating the presence of events and changes in the work, couple, family, friends, economic, legal, relationship and health areas during the previous 12 months; (2) Chronic Stress Questionnaire [47]. The questionnaire was open-ended whereby participants could give information about the relative long-lasting problems, conflicts and stressors that they currently have to face. Each answer was evaluated according to its severity, from 1 for not very important to 3 for very important. The total score was obtained by adding the responses of the severity indicated in each of the problems, stressors and threats mentioned; (3) Minor Daily Hassles Questionnaire [47]. Each person was asked to write down the more common everyday demands, irritations and micro-stressors they currently were experiencing. Each answer was evaluated on how much irritation the situation caused, from 1 for “of little importance” to 3 for “very important”. The total score was obtained by adding the responses given to each demand or situation mentioned.

Coping styles were measured by the Spanish version of the Coping Styles Questionnaire [48]. This questionnaire consists of 45 items rated on a 4-point Likert-type scale from 0 “never” to 3 “always”. In the factorization of the individuals in our sample it tests 3 factors: Rational coping style, which comprises 15 items whose internal consistency (Cronbach’s alpha) was 0.84; emotional coping style, consisting of 16 items whose internal consistency was 0.83; and detachment coping style, consisting of 14 items whose Cronbach’s alpha was 0.75.

Self-esteem was assessed using the Spanish version of the York Self-Esteem Inventory [49]. This inventory consists of 51 items which assess evaluative personal, interpersonal, familial, and achievement self-domains. The response format is a 4-point Likert scale ranging from 0 (never) to 3 (always) and higher scores indicate greater levels of self-esteem. In the present sample, the Cronbach alpha for the 51 items of the inventory was 0.95.

Social support was measured by the Social Support Scale [50]. This scale consisted of 12 items assessing emotional, instrumental and advice/guidance perceived social support. The response format is a 4-point Likert scale ranging from 0 (never) to 3 (always) and higher scores indicate greater levels of social support. In the present sample, the Cronbach alpha for the 12 items of the scale was 0.89.

### 2.3. Statistical Analysis

Internal consistency was measured using Cronbach’s alpha coefficient. Comparisons between men and women for the study variables were calculated using Student´s *t*-test, and effect size of the mean differences by using the Cohen’s *d* [51]. Correlation coefficients between the study variables were calculated using the Pearson’s correlation coefficient, except for educational level, which being an ordinal variable, was calculated with Spearman’s Rho. Four-step hierarchical multiple regression analyses were used to determine the relevance of the stress, coping styles, the self-esteem and the social support in men’s and women’s psychological distress. In each regression analysis, the respondents’ age and education, as an ordinal variable with 8 levels (from 0 for basic education to 7 for 5-year university degree) were entered in Model 1 to control their effect; number of life events experienced in the last year, chronic stress and minor daily hassles scores were added in Model 2; coping styles scores were added in Model 3; and the scores on self-esteem and social support were added in Model 4. Due to the large sample size, statistical significance was set at *p* < 0.001. Statistical analyses were conducted with SPSS 22.0 (IBM Corporation, Armonk, NY, USA) software.

## 3. Results

Participants had different educational levels, although the most frequent (38.7% of men and 52.5% of women) were university students, of which many were still studying. While, 36.0% of men and 32.2% of women had secondary education and 25.2% of men and 15.3% of women had basic education only. There was also diversity in their occupation, although most participants were students, which was the case in 39.6% of men and 43.3% of women. 27.1% of men and 22.5% of women performed manual labor, 18.3% of men and 13.2% of women performed non-manual labor, and 9.7% of men and 16.5% had professions that required university studies. Furthermore, 5.3% of women and 4.6% of men were unemployed. Most of the sample (91.7% of men and 85.7% of women) were single and the rest were married or with a partner. Most (97.2% of men and 94.4% of women) had no children and the rest had one or more children.

Table 1 shows the means, the standard deviations and the comparison for men and women in the study variables. Women reported higher scores than men in psychological distress, chronic stress, minor daily hassles, emotional coping style and social support, although most of the effect sizes were small. And men reported higher scores than women in rational and detachment coping styles and in self-esteem.

When analyzing the most frequent sources of stress in emerging adult women and men, it was found that the events and changes during the last 12 months, most frequently cited by non-students, were those related to work, which were reported by 63.6% of men and 63.2% of women. Starting a new job occurred in 37.3% of men and 40.8% of women; change in working conditions occurred in 37.3% of men and 40.8% of women; job change occurred in 29.9% of men and 29.2% of women; and job loss in 19.4% of men and 19.8% of women. Those who were still students (41.4% of the sample) also reported changes in education, which occurred in 24.4% of men and 22.4% of women. Among these changes, the most frequent was to begin new studies (17.0% of men and 14.6% of women), following by studies change (8.1% of men and 7.5% of women), changes in studies’ conditions (9.0% of men and 7.4% of women), and having failed in their studies (6.9% of men and 6.7% of women).

Changes in the partner relationship were also frequent in the study sample, being reported by 42.2% of men and 42.8% of women. Among these changes, the most frequent were ending the relationship (25.9% of men and 24.93% of women), and beginning a new relationship (23.2% of men and 19.4% of women); and almost 10% (7.9% of men and 10.5% of women) reported that in the last 12 months they had married or started living with their partner; in addition, 15.5% (15.0% of men and 16.0% of women) reported serious discussions with their partner. Other frequent stressors were those related to the family, the most frequent being family discussions (24.3% of men and 30.9% of women), followed by severe illness in a family member (23.1% of men and 26.2% of women), the death of a relative (21.5% of men and 21.3% of women), and the change in the relationship with their parents (18.8% of men and 19.3% of women). They frequently cited the change of place of residence (23.1% of men and 26.3% of women), debts payable (23.3% of men and 19.8% of women) and separation due to geographical circumstances (11.2% of men and 13.2% of women).

Table 2 shows bivariate correlations among the study variables, calculated independently for the group of men and women. The correlation coefficients were similar in women and men, except for the correlations between self-esteem and detachment coping style, and education with self-esteem and social support, which were only statistically significant in women, although the percentage of shared variance was low.

Table 3 shows the main values for hierarchical multiple regression analyses with the psychological distress as the dependent variable for the men, and Table 4 for the women. The results showed that *R* was considerably different from zero at the end of each step. After step 1, with age and education in the equation, *R*^2^ = 0.01, *p* < 0.001 in the men, and *R*^2^ = 0.02, *p* < 0.001 in the women. The change in *R*^2^ from Model 1 to Model 2 made clear the relevance of the stress measures in women and men’s psychological distress. The addition of coping styles measures in Model 3 resulted in an important increment in *R*^2^. The addition of self-esteem and social support in Model 4 produced a statistically significant increment in *R*^2^.

Beta values in Model 4, with all independents variables in the equation, proved that emotional coping style was the variable most associated with psychological distress in both women and men; the second most relevant variable was self-esteem, number of life events experienced during the last year was the third, and social support was the fourth one. Detachment coping style figured as another statistically significant variable in men. In women, chronic stress was the other variable that proved statistically significant. The adjusted *R*^2^ value of 0.42 in the men indicated that 42% of the variability in emerging adulthood men’s psychological distress was predicted by high emotional coping style, lower self-esteem, high number of life events, less social support and less detachment coping style. In the women, *R*^2^ was 0.41 and such 41% of the variability in emerging adulthood women’s psychological distress was predicted by high emotional coping style, lower self-esteem, high number of life events, less social support and high chronic stress.

## 4. Discussion

The importance of stress for psychological distress is well-documented [22,34,52,53], but knowledge is yet to be refined for emerging adulthood, a period of life characterized by major changes, including different social roles [20], that are potential sources of stress. Although the prevalence of psychiatric disorders in this period of the life span is higher than in those in any other range [1], emerging adulthood is also a time of possibilities, where people have an opportunity to transform their lives [2]. According to Arnett [3] (p. 469) “Emerging adulthood is a time of life when many different directions remain possible, when little about the future has been decided for certain, when the scope of independent exploration of life’s possibilities is greater for most people than it will be at any other period of the life course”. Mental and stress-related health among young adults constitute significant public health concerns internationally [23]. Therefore, it is important to carry out research that makes it possible to advance in the knowledge of the variables and factors that promote and limit development, and mental health of emerging adult men and women. 

The current study assessed the relevance of stress, coping styles, self-esteem and perceived social support in the distress of emerging adult women and men. The result of bivariate correlations analyses showed, both in women and men, statistically significant correlation coefficients between psychological distress and three measures of stress (number of life events experienced during the last year, chronic stress and minor daily hassles), but the effect size was small. Other research carried out in Spain with adults [34] and the elderly [54] also found that the effect of the extent of the relationship between the number of life events and chronic stress with psychological distress was small. These findings are consistent with literature about the relationship between stressful experiences and psychological difficulties, where modest effect sizes have been found and where it has been argued that stress exposure alone is not sufficient to explain differences in psychological distress [55].

Regression analyses results showed that, when all the variables were included in the equation (Model 4), self-esteem and emotional coping style were more important predictors, despite the number of life events was a relevant predictor of psychological distress. The results showed that the most salient predictor of emergent adult women’s and men’s psychological distress was emotional coping style. This finding is consistent with other research which has found associations between psychological distress and emotion focused coping [34,54,56,57]. In the present study, the second most important predictor of psychological distress, in both men and women, was lower self-esteem. This finding converges with and expands on existing literature about relationship between self-esteem and mental health symptoms [37,38] and the role of self-esteem as a mechanism by which stressors impact mental health [28,29,35,36]. The regression analyses also showed that low social support was associated with women and men’s psychological distress, although the effect size was small. This finding is consistent with literature about relationship between social support and mental health [4,32,33,34,58].

The second aim of this study was to examine the existence of differences between emerging adult men and women in distress, stress, coping styles, self-esteem and in perceived social support. Although, most of the effect sizes were small, results showed that women scored higher than men in psychological distress, chronic stress, minor daily hassles, emotional coping style and social support, whereas men scored higher than women in rational and detachment coping styles and in self-esteem. However, there were no differences between women and men in the number of life events experienced last year. Detachment coping style is defined “by the feeling of being independent of the events and the emotion associated with it” [48] (p. 623), for example, “Feel independent of the circumstances” or “Resolve the issue by not becoming identified with it”. The existence of higher scores in women compared to men in psychological distress is found in research [11,12,14,15,16,17,34,54], and the results of this study show that this also happens in emerging Spanish adult women and men. Also, in other research carried out in Spain with adults and elderly people, it has been found that women reported more chronic stress than men [34,54,59] and they did not differ in the number of life events experienced [34,54]. The results of this work in gender differences in coping styles are consistent with, and extend to emerging adulthood, results found in research conducted in Spain with adults and elderly people [34,54,59], where it has been found that women scored higher than men in emotional coping, whereas men scored higher than women in rational coping.

Gender is recognized as an important social determinant of health [41,42,43], and although gender differences have been found in all study variables, except the number of life events experienced during the last year, the most important predictors of emerging adult men and women’s psychological distress were the same variables. The exception was the detachment coping style that was negatively associated with psychological distress only in the male group. Although, chronic stress was only statistically significantly associated with psychological distress in women, this was because statistical significance was set at *p* < 0.001, but Beta weights were very similar for women and men (β = 0.07, *p* < 0.001 in women and β = 0.06, *p* = 0.002 in men). 

Although, the research does not elucidate the exact origin of the differences between women and men, the results indicate that differences are related to gender roles and biases. Therefore, the results have shown that women have higher mean scores in emotional coping style than men and that men have higher mean scores in rational and detachment coping styles than women, this difference could be due to the internalization of gender stereotypes that associate men with reason and women with emotion. Despite the fact that such stereotypes portray women and men as opposites and complementary [60], with the exception of detachment coping style, the significant predictors of psychological distress were practically identical for women and men, suggesting that they both react in a very similar way in terms of psychological distress.

Some limitations to the study should be mentioned. Firstly, this is a cross-sectional design; therefore, no cause-effect inferences can be made. Although, longitudinal studies proved definitively that stressful events consistently played a causal role in the generation of a wide variety of physical and psychological outcome [55], conducting a longitudinal study would make it possible, for example, to explain cause-effect relationships between variables that have been shown to be closely related, such as self-esteem with emotional and rational coping styles. In addition, conducting a longitudinal study would clarify the evolution of the stress-health process through the period of emerging adulthood. Second, the sample, although large, is a convenience sample. Future studies should be conducted with a random sample of emerging adults to ensure that it is a representative sample of the population. Third, all the variables have been measured by self-report, a method that is subject to biases due to factors such as social desirability and/or memory distortions. Fourth, all the participants lived in Spain, which may restrict the generalization of results with respect to other countries, since it has been recognized that there are connections between society and culture with the exposure to and meaning of stressors, access to personal and social resources, and mental health outcomes [29,35]. Finally, in order to increase the percentage of variance explained, in addition to self-esteem and social support, other variables that have shown to be relevant in the stress-health process could have been included as predictors, such as, mastery [35,36].

## 5. Conclusions

Findings from the present study supported the existence of a relationship between stress and coping styles with psychological distress in emerging adults. Although there were some differences between women and men in psychological distress, chronic stress, minor daily hassles, emotional coping style, detachment coping styles, self-esteem and social support, the main predictors of psychological distress were the same for emerging adult women and men, the most important being high emotional coping style, lower self-esteem, and high number of life events. Mental health during emerging adulthood has important implications for identity exploration, relational behaviors and future mental health outcomes and behaviors, and interventions for psychological distress may reduce these serious consequences. The results of this research are relevant to healthcare professionals interested in improving mental health and preventing mental disorders in this important period of life.

## Figures and Tables

**Table 1:** Means (*M*), standard deviations (*SD*) and comparisons for men and women for the study’ variables.

Study Variables	Men (*n* = 2408)		Women (*n* = 2408)		*t*_(4814)_	*d*-Value
	M	SD	M	SD		
Psychological distress	18.46	10.39	22.17	11.52	−11.73 *	0.34
Number of life events	3.54	2.66	3.54	2.65	−0.08	0.00
Chronic stress	4.97	4.02	5.61	4.26	−5.34 *	0.15
Minor daily hassles	4.43	3.58	5.26	3.85	−7.82 *	0.22
Rational coping style	26.84	6.66	24.80	6.28	10.90 *	0.32
Emotional coping style	15.02	6.70	16.49	6.58	−7.66 *	0.22
Detachment coping style	15.84	5.26	14.29	5.14	10.35 *	0.30
Self-esteem	108.27	20.44	104.24	21.20	6.71 *	0.19
Social support	28.75	6.12	29.94	5.82	−6.91 *	0.20

*d*-value = Cohen’s *d*. * *p* < 0.001.

**Table 2:** Correlations among the study variables for the groups of men and women. Correlations for men are above the diagonal; correlations for women are below the diagonal.

	1	2	3	4	5	6	7	8	9	10	11
1. Age		0.09 *	−0.06	0.09 *	0.02	0.06 *	0.08 *	−0.12 *	0.06	0.14 *	0.08 *
2. Educational level	0.17 *		−0.05	−0.10 *	0.09 *	0.09 *	0.17 *	−0.07 *	−0.04	0.06	0.06
3. Psychological distress	−0.04	−0.11 *		0.24 *	0.19 *	0.13 *	−0.24 *	0.59 *	0.08 *	−0.55 *	−0.31 *
4. Number of life events	0.07 *	−0.10 *	0.27 *		0.22 *	0.18 *	−0.02	0.18 *	0.05	−0.12 *	−0.11 *
5. Chronic stress	0.07 *	0.08 *	0.22 *	0.26 *		0.54 *	−0.02	0.14 *	−0.01	−0.14 *	−0.15 *
6. Minor daily hassles	0.03	0.11 *	0.13 *	0.13 *	0.47 *		0.02	0.11 *	−0.01	−0.07	−0.08 *
7. Rational coping style	0.06	0.13 *	−0.24 *	0.00	−0.01	0.02		−0.22 *	0.16 *	0.48 *	0.22 *
8. Emotional coping style	−0.09 *	−0.13 *	0.58 *	0.17 *	0.18 *	0.11 *	−0.28 *		0.28 *	−0.69 *	−0.32 *
9. Detachment coping style	0.03	−0.09 *	0.04	0.03	0.01	−0.02	0.26 *	0.15 *		−0.15 *	−0.05
10. Self−esteem	0.08 *	0.12 *	−0.52 *	−0.11 *	−0.15 *	−0.06	0.51 *	−0.68 *	−0.02		0.36 *
11. Social support	0.02	0.15 *	−0.31 *	−0.17 *	−0.14 *	0.04	0.20 *	−0.33 *	−0.05	0.34 *	

* *p* < 0.001.

**Table 3:** Summary of the hierarchical regression with the psychological distress as the dependent variable for the men.

Measure	Model 1		Model 2		Model 3		Model 4	
	β	*t*-Value	β	*t*-Value	β	*t*-Value	β	*t*-Value
Age	−0.06	−2.99	−0.09	−4.36 *	−0.01	−0.50	0.01	0.30
Education	−0.05	−2.38	−0.04	−1.88	0.01	0.68	0.01	0.10
Number of life events			0.21	10.26 *	0.13	7.62 *	0.12	7.58 *
Chronic stress			0.14	5.81 *	0.08	4.13 *	0.06	3.06
Minor daily hassles			0.02	1.08	0.01	0.33	0.01	0.60
Rational coping style					−0.11	−6.45 *	−0.02	−0.84
Emotional coping style					0.55	30.82 *	0.39	16.96 *
Detachment coping style					−0.06	−3.68 *	−0.07	−4.26 *
Self-esteem							−0.23	−9.38 *
Social support							−0.08	−4.44 *
*R* ^2^	0.01		0.09		0.39		0.42	
Adjusted *R*^2^	0.01		0.08		0.39		0.42	
*R*^2^ Change	0.01		0.08		0.30		0.03	
ANOVA (*F*-value, df)	7.87 (22,405) *		45.58 (22,405) *		191.99 (22,405) *		173.33 (22,405) *	

β = Standardized regression coefficient. *R*^2^ = percentage of explained variance. * *p* < 0.001; *t*-value = Student’s *t*.

**Table 4:** Summary of the hierarchical regression with the psychological distress as the dependent variable for the women.

Measure	Model 1		Model 2		Model 3		Model 4	
	β	*t*-Value	β	*t*-Value	β	*t*-Value	β	*t*-Value
Age	−0.02	−1.10	−0.05	−2.66	−0.01	−0.08	0.00	−0.01
Education	−0.13	−6.19 *	−0.11	−5.63 *	−0.04	−2.23	−0.03	−1.85
Number of life events			0.22	10.71 *	0.16	9.26 *	0.15	8.90 *
Chronic stress			0.16	7.21 *	0.08	4.38 *	0.07	3.54 *
Minor daily hassles			0.04	1.85	0.02	1.18	0.03	1.57
Rational coping style					−0.09	−5.07 *	−0.01	−0.46
Emotional coping style					0.51	28.76 *	0.38	16.99 *
Detachment coping style					−0.02	−1.48	−0.03	−1.98
Self−esteem							−0.20	−8.25 *
Social support							−0.08	−4.68 *
*R* ^2^	0.02		0.12		0.39		0.41	
Adjusted *R*^2^	0.02		0.11		0.38		0.41	
*R*^2^ Change	0.02		0.10		0.27		0.02	
ANOVA (*F*-value, df)	21.36 (22,405) *		63.67 (22,405) *		189.17 (22,405) *		167.80 (22,405) *	

β = Standardized regression coefficient. *R*^2^ = percentage of explained variance. * *p* < 0.001; *t*-value = Student’s *t*.
